# Discovery of lipid biomarkers correlated with disease progression in clear cell renal cell carcinoma using desorption electrospray ionization imaging mass spectrometry

**DOI:** 10.18632/oncotarget.26706

**Published:** 2019-03-01

**Authors:** Keita Tamura, Makoto Horikawa, Shumpei Sato, Hideaki Miyake, Mitsutoshi Setou

**Affiliations:** ^1^ Department of Cellular and Molecular Anatomy, Hamamatsu University School of Medicine, Hamamatsu, Shizuoka, Japan; ^2^ Department of Urology, Hamamatsu University School of Medicine, Hamamatsu University School of Medicine, Hamamatsu, Shizuoka, Japan; ^3^ International Mass Imaging Center, Hamamatsu University School of Medicine, Hamamatsu, Shizuoka, Japan; ^4^ Preeminent Medical Photonics Education and Research Center, Hamamatsu, Shizuoka, Japan; ^5^ Department of Anatomy, The University of Hong Kong, Hong Kong, China

**Keywords:** clear cell renal cell carcinoma, desorption electrospray ionization imaging mass spectrometry, lipid, biomarker, disease progression

## Abstract

Clear cell renal cell carcinoma (ccRCC) often results in recurrence or metastasis, and there are only a few clinically effective biomarkers for early diagnosis and personalized therapy. Metabolic changes have been widely studied using mass spectrometry (MS) of tissue lysates to identify novel biomarkers. Our objective was to identify lipid biomarkers that can predict disease progression in ccRCC by a tissue-based approach. We retrospectively investigated lipid molecules in cancerous tissues and normal renal cortex tissues obtained from patients with ccRCC (*n* = 47) using desorption electrospray ionization imaging mass spectrometry (DESI-IMS). We selected eight candidate lipid biomarkers showing higher signal intensity in cancerous than in normal tissues, with a clear distinction of the tissue type based on the images. Of these candidates, low maximum intensity ratio (cancerous/normal) values of ions of oleic acid, *m/z* 389.2, and 391.3 significantly correlated with shorter progression-free survival compared with high maximum intensity ratio values (*P* = 0.011, *P* = 0.022, and *P* < 0.001, respectively). This study identified novel lipid molecules contributing to the prediction of disease progression in ccRCC using DESI-IMS. Our findings on lipid storage may provide a new diagnostic or therapeutic strategy for targeting cancer cell metabolism.

## INTRODUCTION

Renal cell carcinoma (RCC) originates from the proximal renal tubular epithelial cells and is the most common kidney cancer type; it accounts for over 90% of all kidney cancers [1]. Worldwide, approximately 430,000 new cases of kidney cancer were diagnosed, with more than 140,000 deaths, in 2015 [2]. Although the most effective treatment for localized RCC is surgery, nearly 30% of patients experience disease recurrence after surgical resection [3], and approximately 30% of patients with RCC have metastasis at the time of initial diagnosis [4]. Effective systemic therapies, including vascular endothelial growth factor tyrosine kinase inhibitor, mammalian target of rapamycin inhibitor, and immune checkpoint inhibitor, have been shown to markedly improve the prognosis of metastatic RCC patients [5–10]; however, there is a lack of clear data regarding the selection of optimum agents for individual patients. Therefore, novel biomarkers are required for early diagnosis and personalized therapy for RCC. Moreover, studying molecules that can predict disease progression or prognosis subsequently contributes to the discovery of biomarkers and improves the clinical outcome.

Proteome analysis has become one of the major approaches to identify biomarkers predicting the prognosis of various malignant tumor types, whereas metabolome analysis is a more recent but promising approach in this field. Metabolomics studies have shown that metabolic changes affect the proliferation of cancer cells, cancer cell survival in conditions of nutrient depletion and hypoxia, and the immune system [11]. Lipid-metabolic signatures are one of the characteristic biochemical signatures in cancer [12]. With regard to clear cell RCC (ccRCC), the most common histological subtype that accounts for approximately 70% of various RCC types [13], high levels of cholesterol ester and/or triacylglycerol have been observed in the cancerous tissue [14–16]. Another lipidomic study revealed that ethanolamine-induced upregulation of phosphatidylethanolamine levels inhibits RCC cell proliferation *in vivo* [17].

Compared with conventional mass spectrometry (MS) methods, such as liquid chromatography (LC)-MS, that are widely used for the screening of small biomolecules using tissue lysates, imaging MS (IMS) has the advantage of allowing the direct analysis of the correlations between pathological findings [18]. Matrix-assisted laser desorption ionization IMS (MALDI-IMS) has emerged as a tissue-based approach and has the potential to overcome the disadvantage of conventional MS. We have identified several lipid molecules that are altered in cancerous tissues of triple-negative breast cancer and colorectal cancer using this technique [19, 20]. Although MALDI-IMS covers a wide range of lipid molecules, analyzable molecules depend on the matrix. Recently, desorption electrospray ionization imaging MS (DESI-IMS), a novel molecular anatomy technique, was developed. DESI-IMS is a matrix-free approach that allows the identification of various types of small molecules such as free fatty acids, lipid mediators, phospholipids, and neutral lipids [21], and produces tissue type-specific mass spectra. DESI-IMS has been used as a promising diagnostic tool for a wide variety of malignant tumors, including brain, breast, stomach, liver, colon, rectum, ovarian, bladder, and prostate cancers [22–30]. In addition, DESI-IMS analysis allows grading of tumor subclasses on the basis of lipid profiles. Principal component analysis of DESI-IMS-based phospholipid profile (*m/z* 700–1000) data distinguished between cancerous and normal tissues in RCC [31]. Different ions (*m/z* 788 [PS(36:1)-H]−, 810 [PS(38:4)-H]− and 885 [PI(38:4)-H]−) contributed to this separation. However, this study focused on phospholipids and did not conduct a molecular search in a wide *m/z* range containing free fatty acids and lipid mediators.

In this study, we applied DESI-IMS to analyze a wide range of lipids in specimens from patients with ccRCC to identify lipid biomarkers that can predict disease progression in these patients. We believe that extensive analysis of lipidomic profiles, which are identical within tissue types (cancerous versus normal), is essential to improve the clinical outcome of patients with ccRCC.

## RESULTS

### Clinical and pathological characteristics of the patients

In total, 47 specimens from patients who had received radical or partial nephrectomy were analyzed in this study. The patient characteristics are shown in Table 1. The median follow-up time was 24 (range 1–78) months. During the follow-up period, disease progression was found in 5 cases (10.6%). The Kaplan–Meier survival curve of progression free survival (PFS) for the original population is shown in Supplementary Figure 1.

**Table 1:** Patient characteristics

Patient characteristics	*n* = 47
Gender	
Male	29 (61.7%)
Female	18 (38.3%)
Age median (range) (years)	66 (37–90)
Tumor size median (range) (mm)	45 (11–120)
WHO/ISUP grade	
Grade 1	3 (6.4%)
Grade 2	29 (61.7%)
Grade 3	12 (25.5%)
Grade 4	3 (6.4%)
Microvascular invasion	
negative	15 (31.9%)
positive	32 (68.1%)
Lymphovascular invasion	
negative	41 (87.2%)
positive	6 (12.8%)
Pathological stage	
I	29 (61.7%)
II	1 (2.1%)
III	13 (27.7%)
IV	4 (8.5%)
Progression-free survival rate at 2 years after surgery (95% confidence interval: CI)	0.90 (0.75–0.96)

### DESI-IMS identifies several lipid molecules strongly upregulated in ccRCC tissues

**Figure 1:**
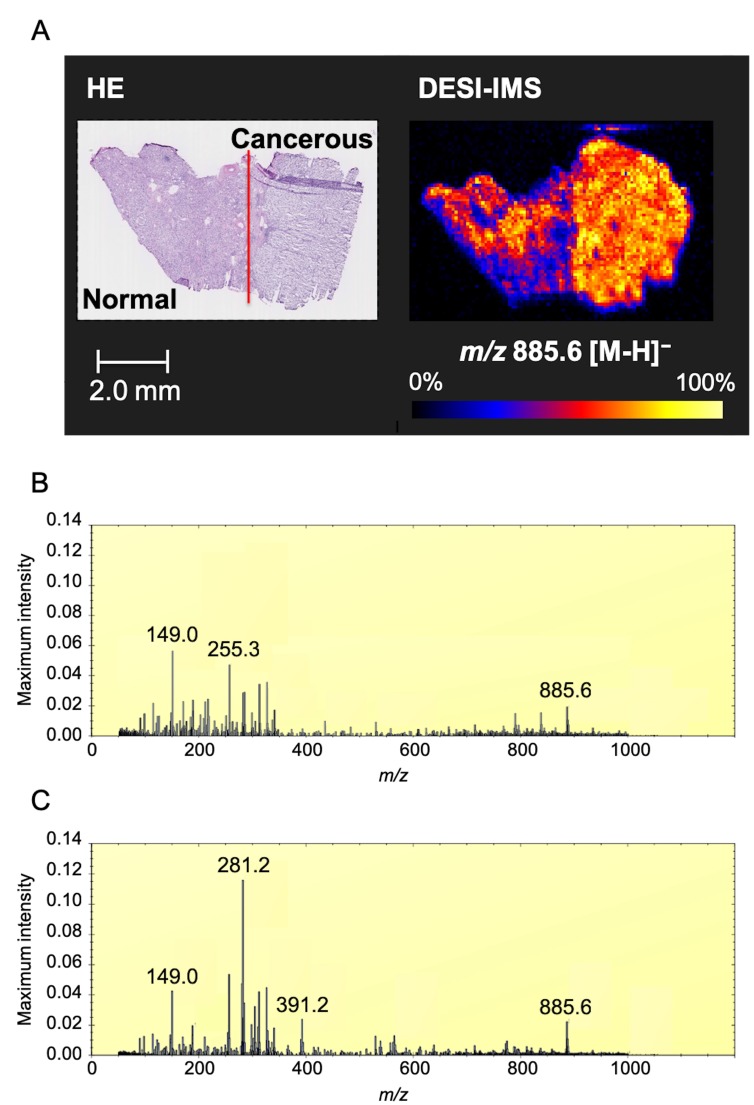
Optimal image of ccRCC by DESI-IMS (**A**) DESI-IMS in negative ion mode showing the signal of ion of *m/z* 885.6 in cancerous tissue and normal tissue distinguished by H&E staining. Molecular ion distribution is shown with normalization to total ion current. High to low ion intensity is shown on a scale from white to black, respectively. Red line shows border between cancerous and normal tissue by pathological analysis. (**B**) Maximum intensity spectrum of normal tissue. (**C**) Maximum intensity spectrum of cancerous tissue. Maximum intensity is normalized to total ion current (TIC).

First, we conducted DESI-IMS on a tissue sample containing the cancerous region and the normal renal cortex region from a single patient with ccRCC. Cancerous and normal tissues were distinguished using hematoxylin and eosin (H&E) staining. DESI-IMS in negative ion mode revealed that the ion of *m/z* 885.6 was highly abundant in the cancerous tissue (Figure 1A). This ion was subsequently identified as glycerophosphoinositol 38:4 [PI(18:0/20:4)], which has been reported as a membrane lipid strongly expressed in breast cancer cells [32].

The maximum intensity peaks for cancerous and normal tissues were normalized to total ion current (TIC) (Figure 1B and 1C). The ion of *m/z* 281.2 was the most abundant in the cancerous tissue. The ions of *m/z* 149.0, 255.2, and 325.2 were the most abundant in the normal tissue.

### Exploration of candidate biomarker lipids of ccRCC

**Figure 2:**
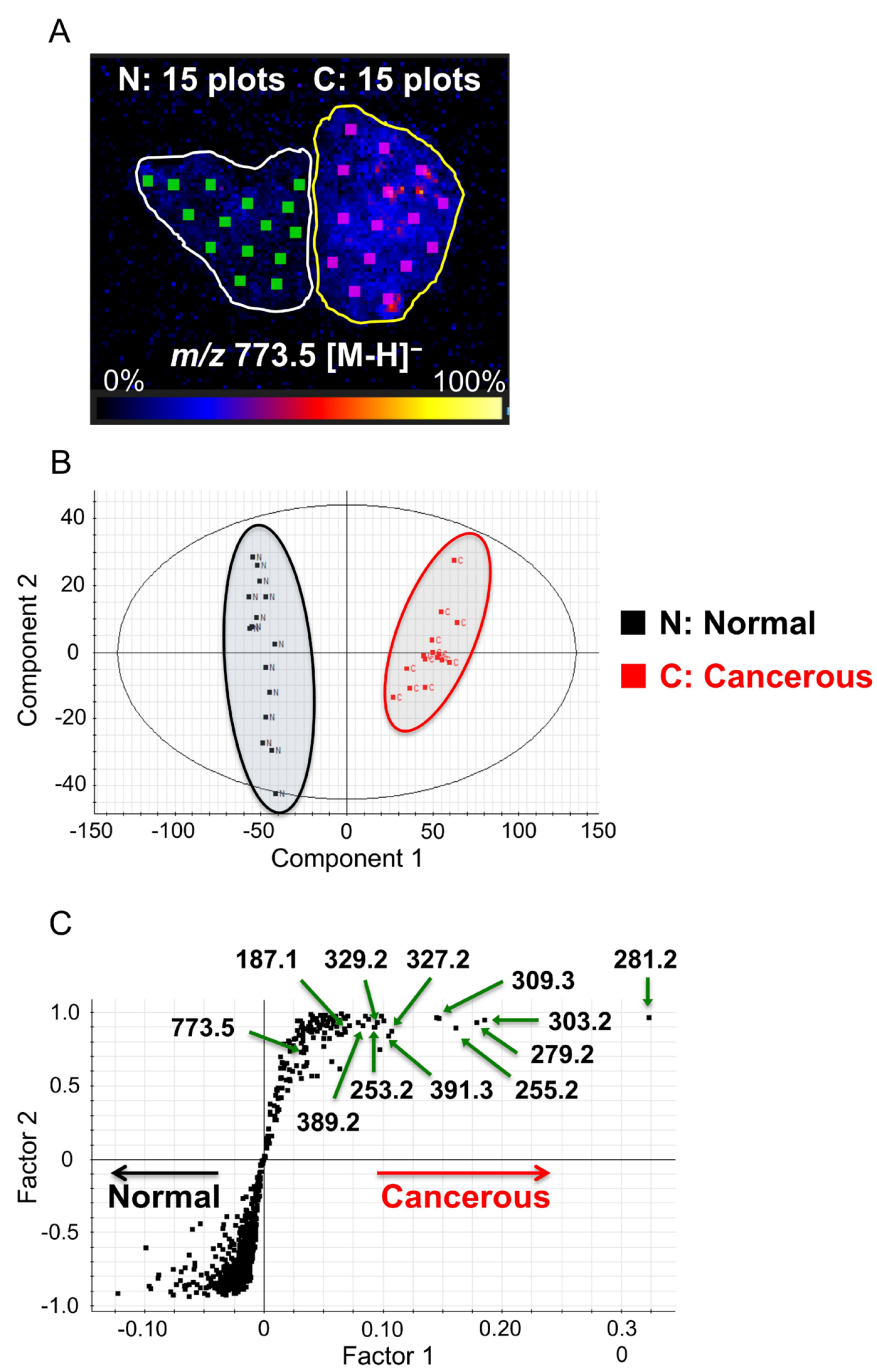
Selection of biomarkers on the basis of orthogonal partial least squares discriminant analysis (OPLS-DA) (**A**) Image showing 15 random regions of interest (ROIs), 0.9 mm^2^ in size, in cancerous (**C**) and normal (N) tissues subjected to DESI-IMS. High to low ion intensity is shown on a scale from white to black, respectively. (**B**) Component 1 (x-axis) and component 2 (y-axis) are the two variables that most strongly contribute to the separation of the data. (C) Covariance factor 1 and correlation factor 2 loadings from two-class OPLS-DA model (C vs. N) are shown in S-Plot format.

Based on the above results, we screened for biomarker candidates that are increased in ccRCC cancerous tissue. We randomly selected fifteen areas of the same size as ROIs in each cancerous and normal tissue, as shown in Figure 2A. We subjected DESI-IMS data for these ROIs to orthogonal projections to latent structures discriminant analysis (OPLS-DA), which clearly distinguished the cancerous tissue from the normal tissue (Figure 2B). The upper right quadrant of the S-plot in Figure 2C shows those components that were elevated in the cancerous tissue, whereas the lower left quadrant shows those components that were elevated in the normal tissue. The measured intensities were based on the average of the measured values for each point in each tissue. This analysis showed that fatty acids that were subsequently identified tended to be more abundant in cancerous tissues than in normal tissues.

### Identification of lipid molecules in ccRCC tissue

Verification of the identified lipid molecules was achieved by LC-MS/MS analyses and mass lock correction on MassLynx. We confirmed *m/z* 885.6 as PI(18:0/20:4) (glycerophosphoinositol 38:4) by LC-MS/MS. We also identified *m/z* 773.5, which was highly expressed in cancerous tissue as PG(18:1/18:1) (glycerophosphoglycerol 36:2). LC-MS/MS results are shown in Supplementary Table 1. Next, mass lock correction was performed using *m/z* 885.5493 (theoretical mass) as a mass calibrator for identifying fatty acids. These molecular assignments were confirmed by database search. Tentative assignments of fatty acids are shown in Table 2. However, we could not assign *m/z* 381.2 and 391.3 to molecules in these analyses. Measured and theoretical mass values from database search, redundancies, and possible hits are shown in Supplementary Tables 2 and 3.

**Table 2:** Tentative assignments of important lipids

Observed mass (HDI)	Measured mass (mass lock correction)	Theoretical mass	Mass error (ppm)	Tentative ion attribution	Elemental formula
187.1	187.0976	187.0970	3.2	azelaic acid	C_9_H_15_O_4_^−^
253.2	253.2174	253.2168	2.4	FA(16:1)	C_16_H_29_O_2_^−^
255.2	255.2330	255.2324	2.4	FA(16:0)	C_16_H_31_O_2_^?−^
279.2	279.2330	279.2324	2.3	FA(18:2)	C_18_H_31_O_2_^−^
281.2	281.2486	281.2481	1.8	FA(18:1)	C_18_H_33_O_2_^−^
303.2	303.2329	303.2324	1.6	FA(20:4)	C_20_H_31_O_2_^−^
309.3	309.2799	309.2794	1.6	FA(20:1)	C_20_H_37_O_2_^−^
327.2	327.2333	327.2324	2.8	FA(22:6)	C_22_H_31_O_2_^−^
329.2	329.2476	329.2481	–1.5	FA(22:5)	C_22_H_33_O_2_^−^
389.2	389.2462	389.2481	–4.9	−	C_27_H_33_O_2_^?−^
		389.2457	1.3	−	C_20_H_38_O_5_P^−^
391.3	391.2586	391.2613	–6.9	−	C_20_H_40_O_5_P^−^
		391.2555	7.9	−	C_27_H_36_P^?−^

Each measured mass was corrected by mass lock of m/z 885.5493 (theoretical mass) as a mass calibrator. FA = fatty acid. Abbreviations: FA, fatty acid. FA(16:1): palmitoleic acid, FA(16:0): palmitic acid, FA(18:2): linoleic acid, FA(18:1): oleic acid, FA(20:4): arachidonic acid, FA(22:6): docosahexaenoic acid (DHA), FA(22:5): docosapentaenoic acid (DPA).

### Selection and validation of eight novel lipid biomarker candidates

We next performed DESI-IMS on sections of all 47 patients and selected candidate biomarkers according to the selection criteria. We selected the ions for which the maximum intensity ratio (MIR) values of cancerous/normal tissue in specimens from the same patient were among the 100 highest MIR values. Additionally, we narrowed down to candidate ions for which the median maximum intensity in cancerous tissues was >1.5 times higher than that in normal tissues. Finally, we selected eight candidate biomarkers having a clearly confirmed peak distribution in the tissue through HDI software.

**Figure 3:**
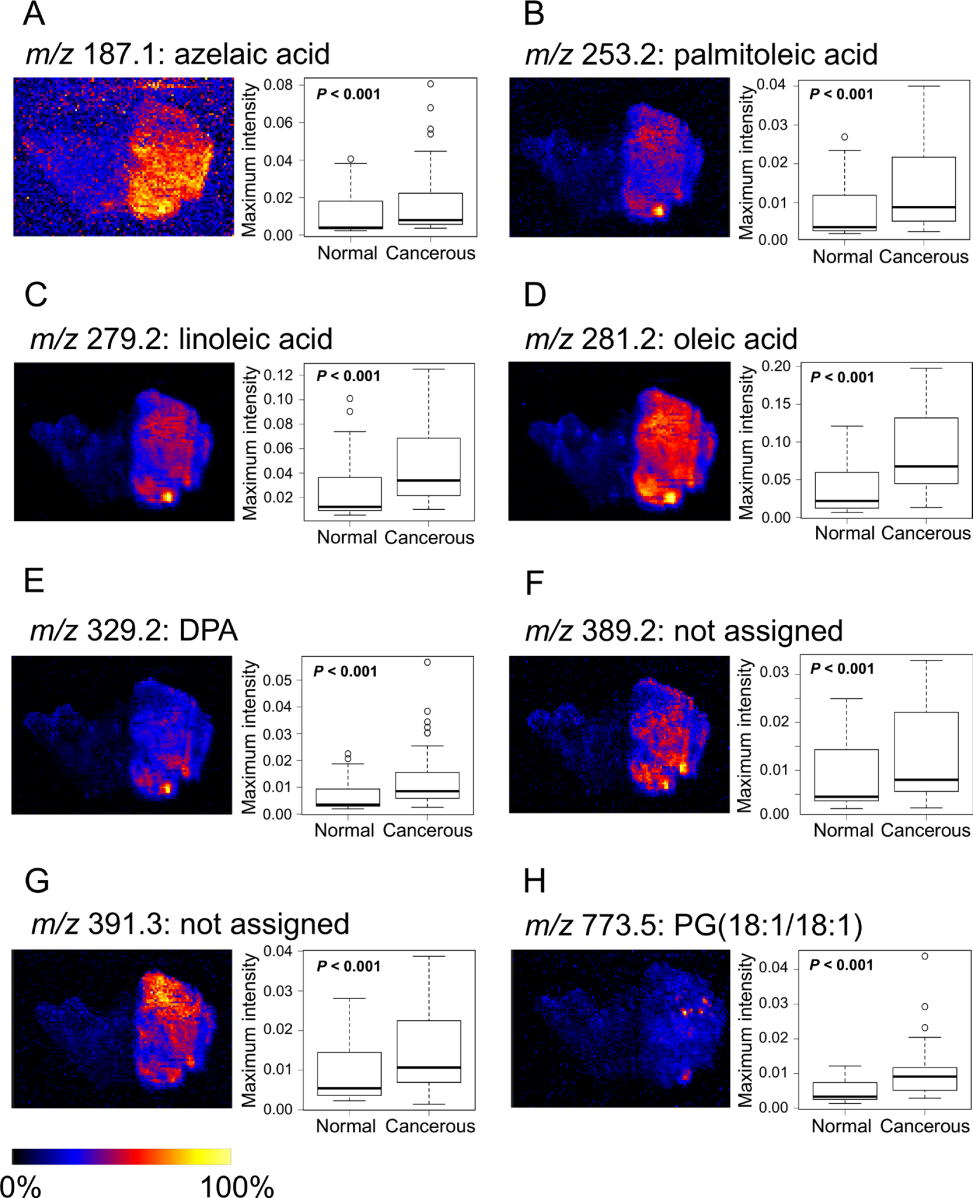
Images and boxplots of maximum intensities for candidate biomarkers by DESI-IMS (**A**) *m/z* 187.1: azelaic acid, (**B**) *m/z* 253.2: palmitoleic acid, (**C**) *m/z* 279.2: linoleic acid, (**D**) *m/z* 281.2: oleic acid, (**E**) *m/z* 329.2: DPA, (**F**) *m/z* 389.2 (not assigned), (**G**) *m/z* 391.3 (not assigned), (**H**) *m/z* 773.5: PG(18:1/18:1). Images of the eight candidate biomarkers contain cancerous and normal tissues. High to low ion intensity is shown on a scale from white to black, respectively. Boxplots show tissue type (x-axis) and maximum intensity normalized to TIC (y-axis). *P*-values were determined using the log-rank test. Each box represents the 25th and 75th percentiles (Q1 and Q3), and the band in the middle indicates the 50th percentile (Q2). The upper and the lower whiskers represent Q3 + 1.5 IQR and Q1−1.5 IQR. The other points represent outliers.

The ions of biomarker candidates were *m/z* 187.1 (azelaic acid), 253.2 [FA(16:1)] (palmitoleic acid), 279.2 [FA(18:2)] (linoleic acid), 281.2 [FA(18:1)] (oleic acid), 329.2 [FA(22:5)] (docosapentaenoic acid: DPA), 389.2 (not assigned), 391.3 (not assigned), and 773.5 [PG(18:1/18:1)] (glycerophosphoglycerol 36:2). Optimal images and box plots of maximum intensities for each candidate marker by DESI-IMS are shown in Figure 3. The maximum intensities of the candidate biomarkers were significantly higher in cancerous than in normal tissues from all patients (*P* < 0.001). The images by DESI-IMS allowed a clear distinction of the cancerous from the normal tissues.

### Correlation of candidate biomarkers from DESI-IMS findings with clinical variables

**Figure 4:**
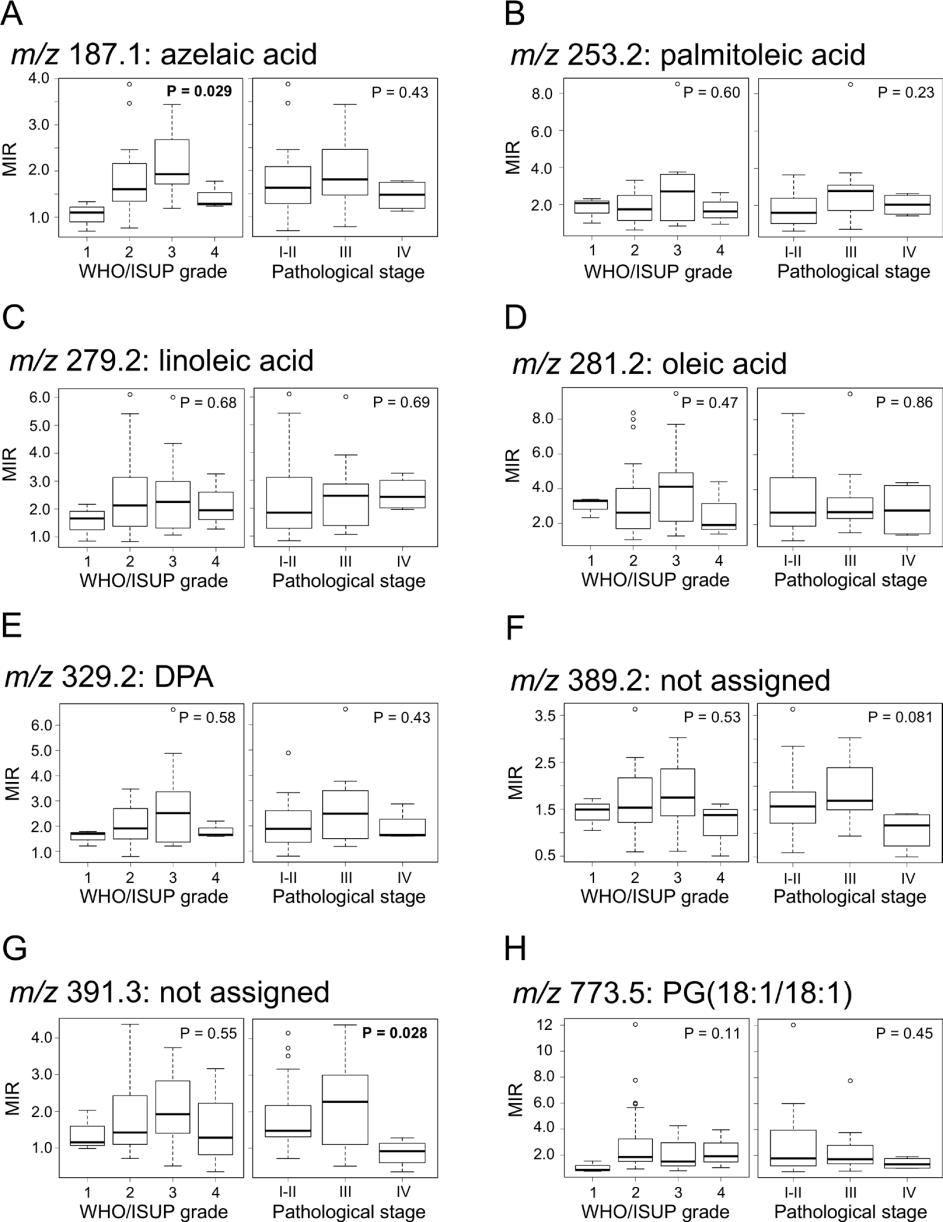
Correlation between candidate biomarkers and tumor grade or pathological stage (**A**) *m/z* 187.1: azelaic acid, (**B**) *m/z* 253.2: palmitoleic acid, (**C**) *m/z* 279.2: linoleic acid, (**D**) *m/z* 281.2: oleic acid, (**E**) *m/z* 329.2: DPA, (**F**) *m/z* 389.2 (not assigned), (**G**) *m/z* 391.3 (not assigned), (**H**) *m/z* 773.5: PG(18:1/18:1). Boxplots show WHO/ISUP grade or pathological stage (x-axis) and MIR values (y-axis). *P*-values were determined using Kruskal-Wallis test. Each box represents the 25th and 75th percentiles (Q1 and Q3), and the band in the middle indicates the 50th percentile (Q2). The upper and the lower whiskers represent Q3 + 1.5 IQR and Q1−1.5 IQR. The other points represent outliers.

We analyzed the correlation between MIR values and tumor grade or pathological stage. The MIR value of azelaic acid significantly correlated with tumor grade (*P* = 0.029), but there was no significance in comparison between each tumor grade (Figure 4A). The MIR value of *m/z* 391.3 significantly correlated with pathological stage (*P* = 0.028), but there was a significance only between pathological stage 1–2 and 4 (Figure 4G). In other candidate biomarkers, the correlation between MIR level and tumor grade or pathological stage was not significant (*P* > 0.05) (Figure 4B–4F and 4H).

### Lipid molecule levels correlate with disease progression of ccRCC

**Figure 5:**
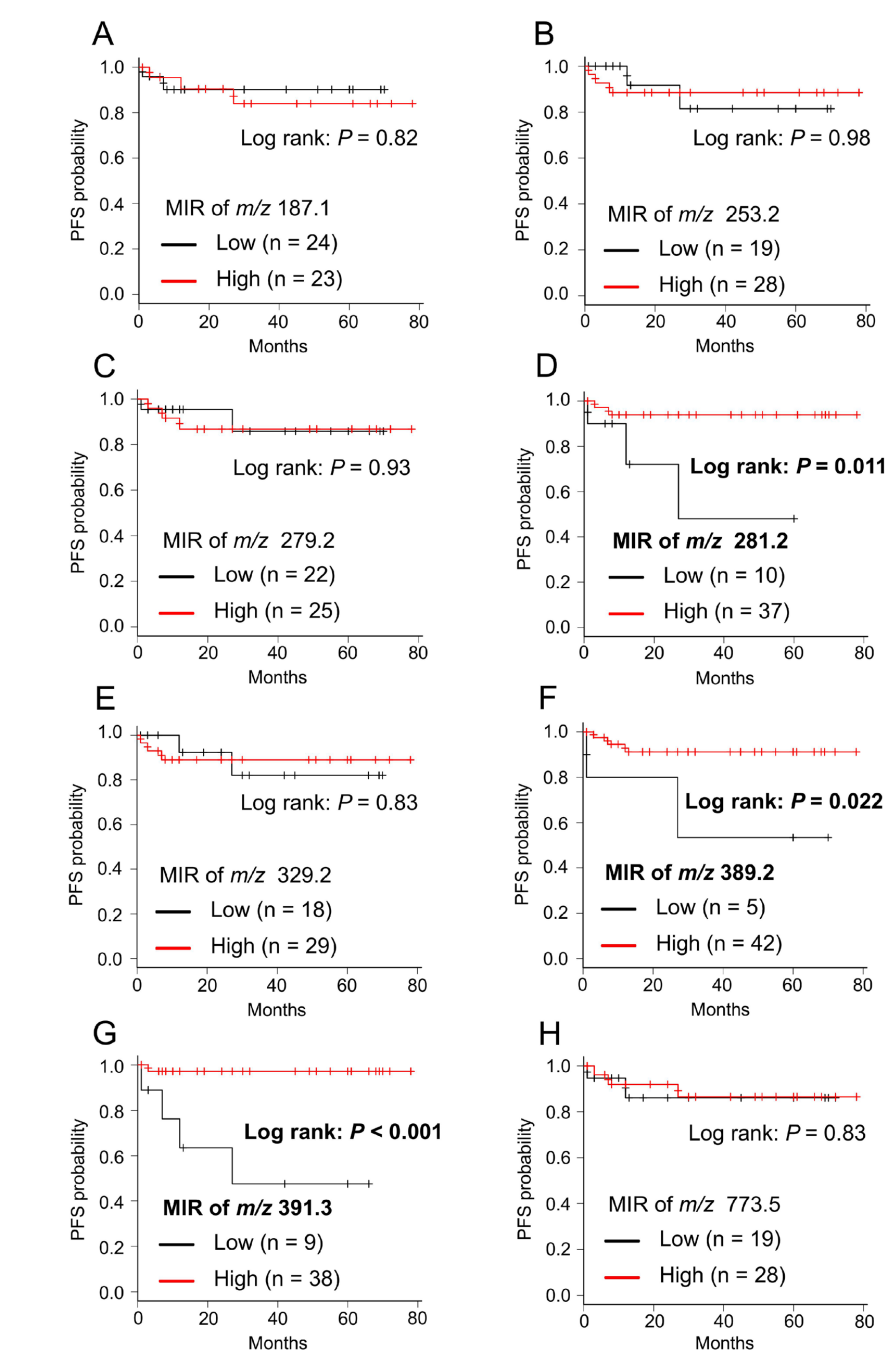
Kaplan–Meier survival curve of PFS for each candidate biomarker (**A**) *m/z* 187.1: azelaic acid, (**B**) *m/z* 253.2: palmitoleic acid, (**C**) *m/z* 279.2: linoleic acid, (**D**) *m/z* 281.2: oleic acid, (**E**) *m/z* 329.2: DPA, (**F**) *m/z* 389.2 (not assigned), (**G**) *m/z* 391.3 (not assigned), (**H**) *m/z* 773.5: PG(18:1/18:1). Cancerous/normal MIR levels were divided in two groups (Low and High) according to cut-off values of each candidate biomarker described in Table 3. *P*-values were determined using the log-rank test.

We used MIR values to analyze progression-free survival (PFS) in ccRCC patients. The MIR cutoff values for each candidate biomarker (azelaic acid, palmitoleic acid, linoleic acid, oleic acid, DPA, *m/z* 389.2, 391.2, and glycerophosphoglycerol 36:2) were determined as 1.72, 1.63, 1.95, 1.63, 1.66, 0.96, 0.98, and 1.61, respectively (Table 3). We performed Kaplan–Meier survival analysis for all candidate biomarkers and examined the correlation between MIR values and disease progression. We found that the MIR values of the ions of oleic acid, *m/z* 389.2, and 391.3 were significantly correlated with disease progression, with low-MIR patients having a significantly shorter PFS (Figure 5D, 5F, and 5G; *P* = 0.011, *P* = 0.022 and *P* < 0.001, respectively). MIR levels of the other candidate biomarkers (azelaic acid, palmitoleic acid, linoleic acid, DPA, and glycerophosphoglycerol 36:2) were not correlated with disease progression (Figure 5A–5C, 5E, and 5H). Sample images of the three lipid biomarkers that correlated with disease progression are shown in Supplementary Figure 2.

**Table 3:** Cutoff values of the cancerous/normal tissue MIRs for each candidate biomarker determined by ROC curve analysis

*m/z*	median MIR of cancerous/normal (IQR)	cutoff value	AUC
187.1	1.66 (1.31–2.12)	1.72	0.51
253.2	2.03 (1.12–2.71)	1.63	0.51
279.2	2.08 (1.34–3.02)	1.95	0.54
281.2	2.70 (1.90–4.50)	1.63	0.58
329.2	1.91 (1.45–2.72)	1.66	0.45
389.2	1.53 (1.26–2.02)	0.96	0.71
391.3	1.53 (1.12–2.44)	0.98	0.79
773.5	1.76 (1.22–3.03)	1.61	0.55

Abbreviations: AUC, area under the ROC curve; MIR, maximum intensity ratio; ROC, receiver operating characteristic.

### Validation of biomarkers predicting disease progression

**Figure 6:**
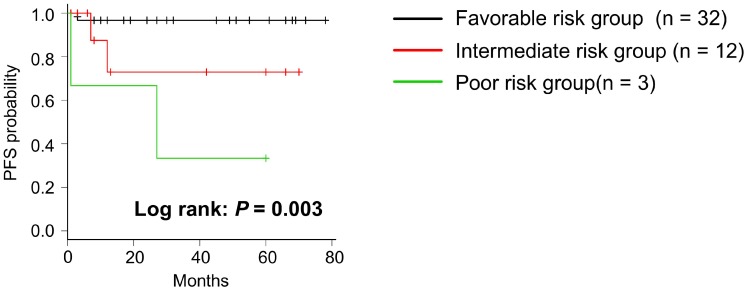
Kaplan–Meier survival curve of PFS in risk stratification Patients were classified into 3 groups according to having a number of risk factors, one of which is a low MIR level in three biomarkers. *P*-value was determined using the log-rank test.

The ions of oleic acid, *m/z* 389.2, and 391.3 are possible novel lipid biomarkers that predict disease progression. We classified patients as having high and low MIR values in each biomarker and whether they had disease progression in Table 4. Sixteen patients had disease progression or low MIR levels of oleic acid, *m/z* 389.2, or 391.3. The other 31 patients had no disease progression and high MIR levels of each biomarker. We defined low MIR levels of each biomarker as a risk factor and included them in the risk stratification, whereby patients with no risk factor were classified as favorable group, those with one to two risk factors were classified as intermediate group, and those with three risk factors were classified as poor risk group. PFS was significantly separated between the three risk groups as shown in Figure 6 (*P* = 0.003). PFS rate at 2 years after surgery was 0.97 (95% CI 0.79 – 0.99) in the favorable group, 0.73 (95% CI 0.28 – 0.93) in the intermediate risk group, and 0.67 (95% CI 0.054 – 0.95) in the poor risk group, respectively.

**Table 4:** List of patients with disease progression or low MIR levels of biomarkers

Patient no.	Progression	Classification of biomarkers		
		Oleic acid	*m/z* 389.2	*m/z* 391.3
5	No	Low	Low	Low
6	Yes	Low	Low	Low
9	No	High	Low	High
10	No	High	High	Low
18	No	High	High	Low
21	Yes	High	High	High
25	Yes	Low	High	Low
27	No	Low	High	Low
30	Yes	High	High	Low
31	No	Low	High	High
37	No	Low	High	High
38	No	Low	High	High
39	No	Low	High	High
43	No	High	High	Low
44	No	Low	Low	High
45	Yes	Low	Low	Low

## DISCUSSION

We evaluated a small cohort of patients for specific ccRCC-related changes in the lipidome in renal tissues by DESI-IMS of cancerous and normal renal cortex samples and their relation with ccRCC progression. The results showed that various types of lipids are present in renal tissues and that fatty acids tended to be more abundant in cancerous than in normal tissues. Based on DESI-IMS data, we selected eight candidate lipid biomarkers, three of which were shown to be associated with disease progression.

The Warburg effect, a process in which cancer cells alter their metabolism, has been widely studied [33, 34]. Cancer cells require energy production and the biosynthesis of several biomolecules for their proliferation and survival [35]. Fatty acid biosynthesis is strongly associated with poor prognosis in various types of malignant tumors [36–38]. Our results from DESI-IMS analysis of ccRCC cancerous and normal tissues demonstrated that various types of fatty acids tended to be more abundant in cancerous than in normal tissues, indicating that cancer cells store fatty acids for energy production. Indeed, activation of lipid storage pathways in ccRCC has been previously shown [39]. In contrast, a recent metabolomics study reported that fatty acid biosynthesis increases during pathogenesis and decreases during ccRCC progression [40]. Moreover, the study showed a decrease in medium-chain fatty acids in late-stage tumors, which was consistent with the observation that the lipid content is decreased in high-grade ccRCC [14]. We consider that lipid storage in cancer cells decreases in high-grade or advanced-stage ccRCC because fatty acid oxidation is strongly activated depending on tumor aggressiveness [41]. In this study, MIR values of the ions of azelaic acid and *m/z* 391.3 were significantly correlated with tumor grade and pathological stage, respectively. However, other candidate biomarkers had no significant correlations. Given the small number of samples used in the study, this observation may be attributed to a lack of statistical basis. However, previous analyses seem to support the notion of low lipid storage of candidate biomarkers in high-grade or advanced-stage ccRCC [14, 40, 41]. Moreover, we found that oleic acid, *m/z* 389.2, and 391.3 showed decreased levels in cancerous tissues from patients showing shorter PFS, although the total lipid storage level increased in all patients. We hypothesize that particular lipid species decrease because they are consumed in energy production, such as fatty acid oxidation, at levels that exceed fatty acid biosynthesis in cancer cells.

Previous studies have used DESI-IMS for lipid profiling of cancerous versus normal tissues for diagnosis or tumor grading; however, they paid less attention to the potential link between lipid profiles and disease progression. Here, we investigated whether the cancerous/normal MIRs of the ions identified by DESI-IMS were quantitatively useful for predicting disease progression. Surprisingly, low MIR levels of ions of *m/z* 281.2 (oleic acid), 389.2 (not assigned), and 391.3 (not assigned) were significantly correlated with disease progression. In breast cancer, it has been shown that oleic acid is a prominent biomarker and is present in both viable and necrotic tumors using DESI-IMS [24]. The currently unassigned ion of *m/z* 391.3 was identified as a biomarker of viable tissue in breast cancer, and lack of this ion, which indicates tumor necrosis, predicts poor prognosis [42]. Similarly, our study showed that the ion of *m/z* 391.3 was abundant in cancerous tissue, whereas it was lower in ccRCC patients with poor progression. Thus, the low cancerous/normal MIR level for the ion of *m/z* 391.3 is considered to reflect poor prognosis.

Some limitations of this study should be acknowledged. First, this was a retrospective study, without external validation. The small sample size, short term, lack of a random cohort, and single-centeredness of this study might have decreased the robustness. Second, we picked up only ions that are more abundant in cancerous tissues than in normal tissues as candidate biomarkers; molecules that are less abundant in cancerous than in normal tissues can also provide candidate biomarkers to predict progression. Finally, patients with one risk factor, for example a low MIR level in the three biomarkers (oleic acid, *m/z* 389.2, and 391.3) predicting disease progression, might not always have the other two risk factors. This might be caused by the difference in the disposition of each biomarker and determination of cut-off values of MIRs without an optimized sample size. Additionally, we needed to identify the ions of *m/z* 389.2 and 391.2, but we could not do this with LC-MS/MS and mass lock correction.

In conclusion, we performed DESI-IMS to investigate various types of lipid molecules expressed in renal tissues to identify biomarkers that can predict disease progression in patients with ccRCC. The study revealed that fatty acids tended to be more abundant in cancerous tissues. Specifically, eight lipid molecules were elevated in cancerous tissues not only in terms of associated ion intensity but also in terms of distinguishing between cancerous and normal tissues by confirming the image in ccRCC. Low cancerous/normal MIRs of ions of oleic acid, *m/z* 389.2 (not assigned), and 391.3 (not assigned) were shown to be significantly associated with disease progression. These findings on lipid storage provide new insights into tumor severity and prognosis in patients with ccRCC and may aid in the development of a diagnostic or therapeutic strategy for targeting cancer cell metabolism.

## MATERIALS AND METHODS

### Patients and samples

This study was approved by the Ethics Committee of Hamamatsu University School of Medicine (IRB No. 16-203) and was performed in accordance with the Declaration of Helsinki. All patients provided informed consent to use their samples in this study.

In total, 47 patients with ccRCC who had received radical or partial nephrectomy between June 1, 2011, and November 31, 2017, at the Hamamatsu University School of Medicine Hospital (Hamamatsu, Japan) were included in this retrospective study. Forty-seven paired samples of cancerous tissues and normal renal cortex tissues (normal tissues) were obtained. Histological diagnosis was determined in accordance with the World Health Organization classification [43]. All tumors were graded by the World Health Organization/International Society of Urological Pathology grading system [44] and were classified according to the pathological tumor-node-metastasis classification [45]. All samples were immediately stored at –80° C after surgical resection.

### Follow-up

Follow up CT scan was performed, basically every three months, to detect disease progression in the first year. Thereafter, patients were followed up every six months. Follow-up time was calculated from the day of surgery to the day of disease progression or was censored at the last follow-up. Tumor recurrence or disease progression was determined as the clinical outcome according to the Response Evaluation Criteria in Solid Tumors (RECIST) version 1.1 [46]: at least a 20% increase in the sum of the smallest measurement diameters of target lesions; and in addition to a relative increase of 20%, the sum must also demonstrate an absolute increase of at least 5 mm (Note: the appearance of one or more new lesions is also considered progression).

### Sample preparation

Each frozen sample was sectioned to a thickness of 10 μm using a cryostat. Sections were mounted onto a glass slide that was placed in a 50-mL tube with drying silica gel and stored at –80° C prior to DESI-IMS measurement [28]. Consecutive 4-μm sections were stained with H&E for histological examination.

### DESI-IMS analysis

All IMS analyses were performed using a Xevo G2XS Quadrupole-Time-Of-Flight Mass Spectrometer (Waters Co., Milford, MA, USA). The glass slides (MICRO SLIDE GLASS, S2111: Matsunami Glass, Kishiwada, Japan) with the 10-μm slices were mounted on a 2D moving stage and the sections were subjected to DESI-IMS in negative ion mode over *m/z* 50 to 1,000, and the 1,000 highest-intensity peaks were extracted. A mixture of methanol and water (Wako Pure Chemical, Osaka, Japan) in a ratio of 98:2 was used as the charged spray solvent and was delivered at a flow rate of 2 μL/min. Parameter settings were: capillary voltage of 5.0 kV, capillary temperature of 120 °C, and nitrogen pressure of 7.0 bar. Each sample was raster-scanned at a velocity of 300 μm/s and a spatial resolution of 100 μm to acquire DESI-IMS.

The maximum intensities of the ions in the *m/z* range of 50–1,000 were obtained from the entire cancerous tissue and the normal tissue regions selected as regions of interest (ROIs) in a High Definition Imaging (HDI) software version 1.35 (Waters) to process the mass spectral data and to construct 2D ion images. Additionally, the spectral data from ROIs on the images were exported to MassLynx (Waters) for mass lock correction.

### OPLS-DA

Average spectral profiles of 15 distinct ROIs in each of the cancerous and normal tissues from a sample were suitably defined through HDI software (guided by pathology). Mass spectra were normalized to TIC. The profiles were analyzed by OPLS-DA using EZ info version 3.0 (Waters).

### Lipid extraction from the cancerous tissue

For lipid extraction, Bligh and Dyer methods with several modifications were used [47]. All reagents in these experiments were purchased from Wako Pure Chemical (Osaka, Japan). The cancerous tissue sample was homogenized in 2250 μL of MeOH/CHCl_3_ (2:1 v/v) with french press and allowed to stand for 10 min, after which 750 μL of CHCl_3_, 1830 μL of H_2_O, and 870 μL of 1 M CH_3_COOH were added to the solution. The final mixture was 8700 μL of 0.1 M MeOH/CHCl_3_/H_2_O (1:1:0.9 v/v/v). After homogenization, the sample was centrifuged for 10 min (1200 × g at 4° C), and 3000 μL of the lower phase was transferred to a glass vial. The lower phase was evaporated under vacuum and dissolved in 100 μL of MeOH for MS analyses.

### Liquid chromatography-MS/MS analyses

Q-Exactive electrospray ionization-MS/MS analyses were performed for identification of some lipid molecules in RCC tissue using a quadrupole orbitrap Fourier transform mass spectrometer (Thermo Scientific, San Jose, CA, USA); 3 μL of derivatized sample was separated on a Thermo Scientific Acculaim 120 column (150 mm × 2.1 mm × 3 μm) (Thermo Scientific). The mobile phase (MP) A consisted of H_2_O/CAN/MeOH (2/1/1 v/v/v), 5 mM HCOONH_4_, 0.1% HCOOH, and MP B consisted of Acetonitrile/Isopropanol (1/9 v/v), 5 mM HCOONH_4_, and 0.1% HCOOH. Gradient conditions were as follows: started at 20% MP B, increased to 80% MP B at 50 min, and then maintained at 80% MP B until 60 min. The overall run time was 60 min. The flow rate was set at 300 μL/min.

Instrument settings: capillary temperature, 250 °C; sheath gas flow rate, 50; auxiliary flow rate, 15; sweep gas flow rate, 0; S-lens RF level, 50; and auxiliary gas heater temperature, 350° C, with a spray voltage of 3.5 kV in positive mode and 2.5 kV in negative mode. Full-MS mode at 70,000 resolution with an AGC target of 1e6 was used for the quantification runs. A top 10 ddMS2 method was applied for the identification runs using 17,500 MS2 resolution as well as normalized collision energies of 15 (+) and 35 (−). Spectral data were recorded in the mass range of 220–2000 *m/z* using profile mode. Xcalibur 2.2 (Thermo Scientific) was used for data acquisition.

### Lipid molecular identification

The identity of several lipids was confirmed by processing of raw data from LC-MS/MS analyses using LipidSearch software (version 4.1). Important lipid molecular formulas and monoisotopic masses were estimated using mass lock correction on MassLynx. Theoretical mass of a molecule identified from LC-MS/MS analyses was established as a lock mass. Tolerance was set at ± 0.005 Da. Identification was then performed by searching against the LIPID Metabolites and Pathways Strategy (LIPID MAPS) database (http://www.lipidmaps.org). Identification was performed by “Search a computationally-generated database of lipid classes or LIPID MAPS structure database (LMSD)” using measured mass from mass lock correction. Mass tolerance was set at ± 0.01 Da. The identity of lipids that could not be identified from LC-MS/MS was confirmed by calculating the possible molecular formulas of each precursor ion from mass lock correction that corresponded to LIPID MAPS database hits.

### Candidate biomarker selection

We selected ions representing candidate biomarkers based on the following criteria: 1) having a MIR value that is among the 100 highest cancerous/normal MIR values; 2) showing a median intensity in cancerous tissues >1.5 times higher than that in normal tissues for targeting highly expressed molecules in cancerous tissues; 3) having a clearly confirmed peak distribution in the tissue through HDI software. Box plots were generated on the basis of the maximum intensities of the ions in cancerous and normal tissues from the 47 patients with ccRCC.

### Statistical analysis

Continuous variables were reported as median value and interquartile range (IQR), as appropriate. Maximum intensities and MIRs of candidate biomarkers were found not to be normally distributed by Kolmogorov–Smirnov tests [48]. Wilcoxon signed-rank tests for the non-parametric variables were used to compare maximum intensities between cancerous and normal tissues [49]. To compare the differences in MIRs among the groups in tumor grade or pathological stage, Kruskal-Wallis test with post-hoc Steel-Dwass tests were used in accordance with normality [50]. The optimal MIR cutoff value was determined by receiver operating characteristic (ROC) curve analysis [51]. The MIR of each candidate biomarker was selected as the independent variable, and survival outcome (progressed/disease-free for PFS) was selected as the dependent variable. The value at which the sum of sensitivity and specificity became the largest was determined as the cutoff value. Further, the area under the ROC curve (AUC) was used to calculate discrimination ability. PFS was estimated using the Kaplan–Meier method, and the log-rank test was applied to compare survival curves [52]. All comparisons were two-sided and were considered statistically significant at *P* < 0.05. All statistical analyses, except OPLS-DA, were performed with EZR (Saitama Medical Center, Jichi Medical University), which is a graphical user interface for R (The R Foundation for Statistical Computing, Vienna, Austria, version 3.4.1) [53].

## SUPPLEMENTARY MATERIALS FIGURES AND TABLES



## Abbreviations

ccRCC: clear cell renal cell carcinoma
DESI-IMS: desorption electrospray ionization imaging mass spectrometry
MIR: maximum intensity ratio
