# Supplementary material for: Discovery of lipid biomarkers correlated with disease progression in clear cell renal cell carcinoma using desorption electrospray ionization imaging mass spectrometry
Source: Oncotarget. 2019 Mar 1;10(18):1688–703. doi: 10.18632/oncotarget.26706 (PMC6422196; doi:10.18632/oncotarget.26706)
Supplement: Supplementary file 1 [file oncotarget-10-1688-s001.pdf]

# Discovery of lipid biomarkers correlated with disease progression in clear cell renal cell carcinoma using desorption electrospray ionization imaging mass spectrometry

## SUPPLEMENTARY MATERIALS

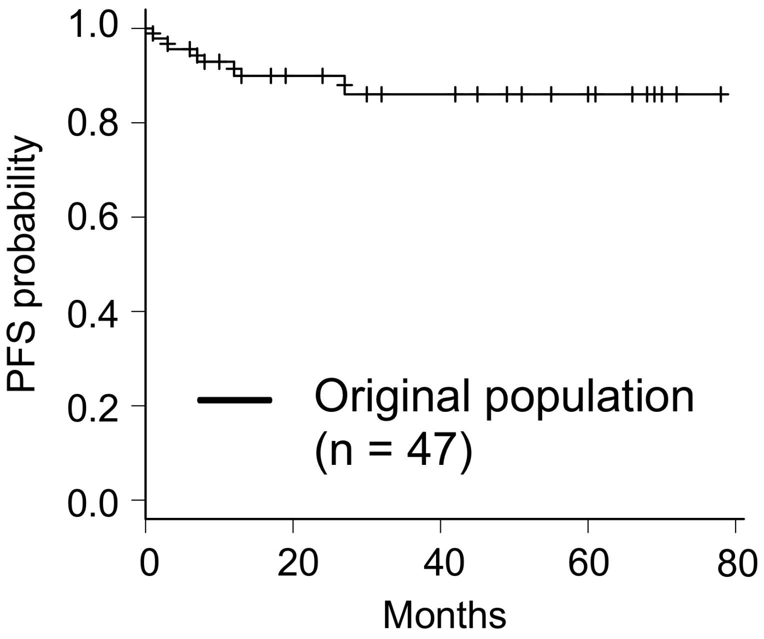


**Supplementary Figure 1: Kaplan–Meier survival curve of PFS for original population.** This survival curve shows observed period (months) (x-axis) and progression-free survival probability (y-axis) in all patients.

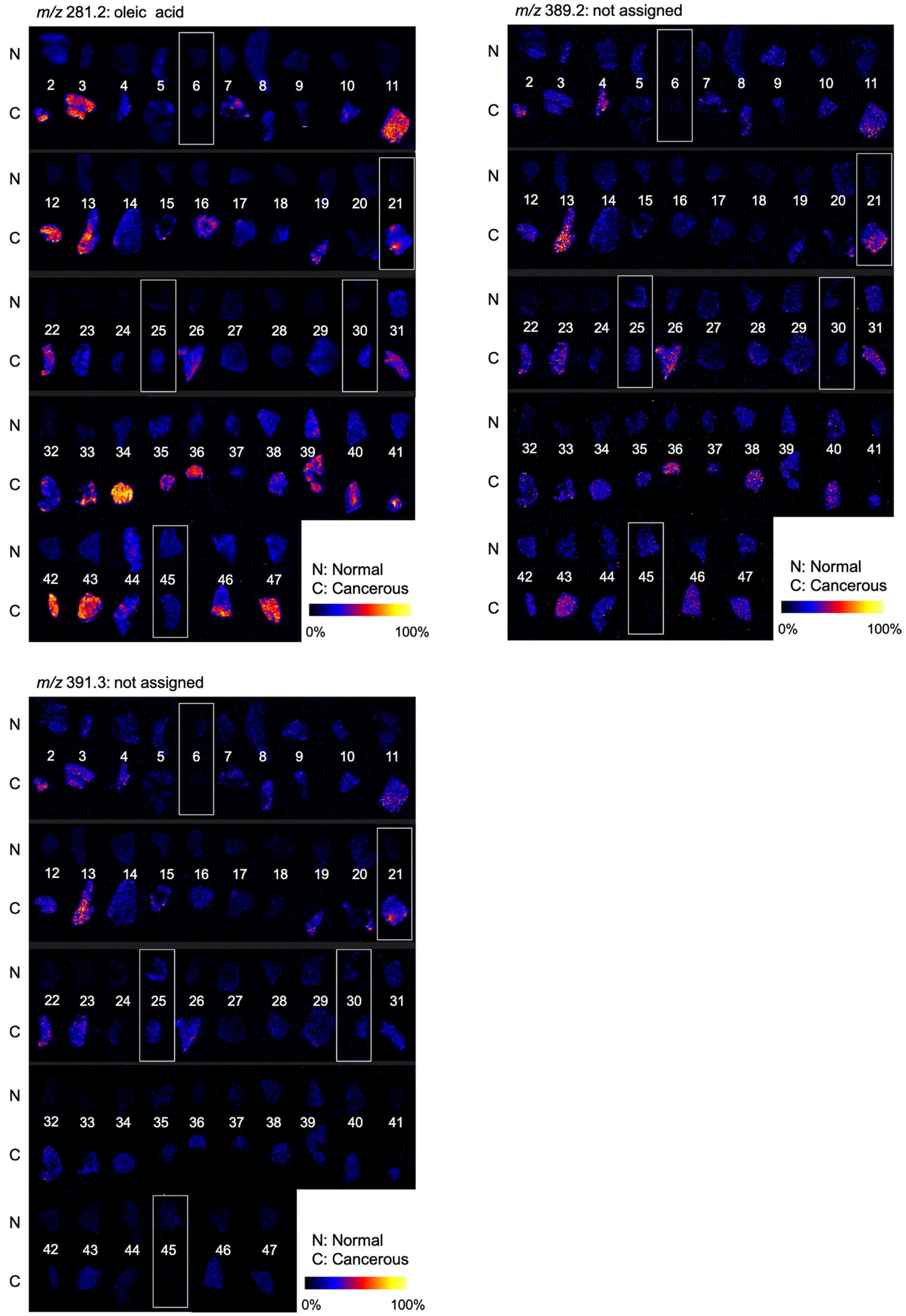


**Supplementary Figure 2: Images showing ions of *m/z* 281.2 (oleic acid), 389.2 (not assigned), and 391.3 (not assigned) identified as biomarkers using DESI-IMS data from all samples except No. 1.** Images for sample No. 1 by DESI-IMS were shown as representative images in the main article. High to low ion intensity was shown on a scale from white to black, respectively. Most cancerous areas were colored in red or blue as compared to normal areas. The samples surrounded with a square box were obtained from patients who showed disease progression.

**Supplementary Table 1: LC-MS/MS data used for identification of glycerophospholipids**

| Observed mass | Theoretical mass | Delta (Da) | Fragment ions | Ion assignments | Elemental Formula |
|---|---|---|---|---|---|
| 773.5341 | 773.5338 | 0.0009 | 152.00, 281.25 | PG(18:1/18:1) | $C_{42}H_{78}O_{10}P^-$ |
| 885.5493 | 885.5499 | 0.0009 | 152.00, 241.01, 259.02, 259.24, 259.24, 283.26 | PI(18:0/20:4) | $C_{47}H_{82}O_{13}P^-$ |

Two important two glycerophospholipids in this study were identified by LC-MS/MS. PG = glycerophosphoglycerol, PI = glycerophosphoinositol.

**Supplementary Table 2: Searching a computationally-generated database of lipid classes**

| Input mass | Matched mass | Delta (Da) | Name | Elemental formula |
|---|---|---|---|---|
| 253.2174 | 253.2173 | 0.0001 | FA(16:1) | $C_{16}H_{29}O_2^-$ |
| 255.2330 | 255.2330 | 0.0000 | FA(16:0) | $C_{16}H_{31}O_2^-$ |
| 279.2330 | 279.2330 | 0.0000 | FA(18:2) | $C_{18}H_{31}O_2^-$ |
| 281.2486 | 281.2486 | 0.0000 | FA(18:1) | $C_{18}H_{33}O_2^-$ |
| 303.2329 | 303.2330 | 0.0001 | FA(20:4) | $C_{20}H_{31}O_2^-$ |
| 309.2799 | 309.2799 | 0.0000 | FA(20:1) | $C_{20}H_{37}O_2^-$ |
| 327.2333 | 327.2330 | 0.0003 | FA(22:6) | $C_{22}H_{31}O_2^-$ |
| 329.2476 | 329.2486 | 0.0010 | FA(22:5) | $C_{22}H_{33}O_2^-$ |
| 389.2462 | 389.2545 | 0.0083 | FA(20:0(OH4,Ep)) | $C_{27}H_{37}O_7^-$ |
| | 389.2545 | 0.0083 | FA(20:0(OH4,Ke)) | $C_{27}H_{37}O_7^-$ |
| 391.2586 | 391.2490 | 0.0096 | FA(23:2(Ke2,Ep)) | $C_{23}H_{35}O_5^-$ |
| | 391.2490 | 0.0096 | FA(23:2(Ke,Ep2)) | $C_{23}H_{35}O_5^-$ |
| | 391.2490 | 0.0096 | FA(23:3(OH,Ep2)) | $C_{23}H_{35}O_5^-$ |
| | 391.2490 | 0.0096 | FA(23:3(OH,Ke2)) | $C_{23}H_{35}O_5^-$ |
| | 391.2490 | 0.0096 | FA(23:3(OH,Ke,Ep)) | $C_{23}H_{35}O_5^-$ |
| | 391.2490 | 0.0096 | FA(23:4(OH2,Ep)) | $C_{23}H_{35}O_5^-$ |
| | 391.2490 | 0.0096 | FA(23:4(OH2,Ke)) | $C_{23}H_{35}O_5^-$ |
| | 391.2490 | 0.0096 | FA(23:5(OH3)) | $C_{23}H_{35}O_5^-$ |

Each input mass is measured mass from mass lock correction described in the main text. The ion of *m/z* 187.0976 did not have any hit on this database search. FA = fatty acid. FA(16:1): palmitoleic acid, FA(16:0): palmitic acid, FA(18:2): linoleic acid, FA(18:1): oleic acid, FA(20:4): arachidonic acid, FA(22:6): docosahexaenoic acid (DHA), FA(22:5): docosapentaenoic acid (DPA).

**Supplementary Table 3: Searching LIPID MAPS structure database (LMSD)**

| Input mass | Matched mass | Delta (Da) | Name | Elemental formula |
|---|---|---|---|---|
| 187.0976 | 187.0976 | 0.0000 | Azelaic acid | $C_9H_{15}O_4^-$ |
| 187.0976 | 187.0976 | 0.0000 | cis- and trans-Ethyl 2,4-dimethyl-1,3-dioxolane-2-acetate | $C_9H_{15}O_4^-$ |
| 187.0976 | 187.0976 | 0.0000 | (+/–)-Ethyl 3-acetoxy-2-methylbutyrate | $C_9H_{15}O_4^-$ |
| 187.0976 | 187.0976 | 0.0000 | (+/–)-Methyl 5-acetoxyhexanoate | $C_9H_{15}O_4^-$ |
| 187.0976 | 187.0977 | 0.0001 | 3-Methylsubericacid | $C_9H_{15}O_4^-$ |
| 391.2586 | 391.2643 | 0.0057 | 5-((3Z,6Z,9Z,12Z,15Z,18Z)-henicosa-3,6,9,12,15,18-hexaen-1-yl)resorcinol | $C_{27}H_{35}O_2^-$ |

This search focused on *m/z* 187.0976, 389.2462, and 391.2586, which could not be identified using a computationally-generated database of lipid classes. The ion of *m/z* 389.2462 did not have any hit on LMSD search.
